# Supplementary material for: Evaluation of awareness, attitudes, and practices towards disaster preparedness among Saudi healthcare professionals: implications for enhancing emergency response and training
Source: PeerJ. 2025 Dec 15;13:e20464. doi: 10.7717/peerj.20464 (PMC12713557; doi:10.7717/peerj.20464)
Supplement: Supplemental Information 2 [file peerj-13-20464-s002.docx]

Data collection form

**Section 1 – Background details**

1. What is your age group in years?

30 or less

31 – 45

46 and above

1. What is your gender?

Male

Female

1. What is your current marital status?

Single

Married

Widow / Divorcee

1. What is your highest education level

Diploma

Bachelor

Masters

Doctorate or equivalent

1. Nationality

Saudi

Expatriates

1. Healthcare professionals (HCPs) category

Physicians

Pharmacists

Lab technicians

Nurses

Others

1. What is your total work experience duration (specify in years)
2. What is your current work setting

Primary health center

General hospital

Specialty hospital

1. Participation in disaster management training

No

Yes

**Section 2:** This section will ask about your awareness of disaster preparedness in local settings. You may choose to answer as yes, no, or not sure

| Awareness | Yes | No | Not sure |
| --- | --- | --- | --- |
| I am familiar with initiatives focused on disaster preparedness |  |  |  |
| I am aware of sources where I can access research or information related to disasters |  |  |  |
| I am knowledgeable about the potential emergency risks in this country (such as natural disasters, embargoes, war, etc.) |  |  |  |
| I am aware of the appropriate contact to reach out to in the event of a disaster emergency (such as the health department) |  |  |  |
| I understand the organizational structures and responsibilities of local and national agencies involved in disaster preparedness. |  |  |  |
| I have enough experience in handling any disaster/emergency‎ |  |  |  |
| Disaster medicine is inherently a systems-focused field involving various responding organizations |  |  |  |
| I am very aware of evacuation protocols from a building in case of any disaster occurrence |  |  |  |
| Overall, my knowledge of disaster management is very high |  |  |  |

**Section 3 - Attitude section:** In this section, you may choose to answer from strongly agree to strongly disagree

| Attitude | Strongly agree | Agree | Neutral | Disagree | Strongly disagree |
| --- | --- | --- | --- | --- | --- |
| I am keen to join disaster medicine education programs that focus on the country’s unique preparedness challenges |  |  |  |  |  |
| I think healthcare professionals play a vital role in community disaster response |  |  |  |  |  |
| I am open to taking on additional responsibilities related to disaster preparedness in my role. |  |  |  |  |  |
| I believe disaster preparedness is important for healthcare professionals. |  |  |  |  |  |
| I feel confident in my ability to respond effectively during a disaster. |  |  |  |  |  |
| I believe that disaster preparedness training should be mandatory for healthcare professionals. |  |  |  |  |  |
| I think my workplace adequately prioritizes disaster preparedness |  |  |  |  |  |
| I believe that effective communication is crucial during a disaster. |  |  |  |  |  |
| I feel supported by my organization in terms of disaster preparedness initiatives. |  |  |  |  |  |
| I am willing to invest time and effort in ongoing disaster preparedness training. |  |  |  |  |  |

**Section 3 - Practice section:** In this section, you may choose to answer from strongly agree to strongly disagree

| Practice | Strongly agree | Agree | Neutral | Disagree | Strongly disagree |
| --- | --- | --- | --- | --- | --- |
| I integrate disaster preparedness practices into my daily routine. |  |  |  |  |  |
| I am confident in my ability to use first aid and CPR techniques during a disaster |  |  |  |  |  |
| I actively contribute to disaster recovery planning efforts in my department |  |  |  |  |  |
| Through sufficient attendance in disaster management training sessions, I feel ready to respond effectively during actual emergencies |  |  |  |  |  |
| I feel comfortable executing evacuation protocols in the event of a disaster |  |  |  |  |  |
| I am familiar with the emergency communication systems in place at my healthcare facility |  |  |  |  |  |
| The training I received during my college courses has equipped me to function effectively in disaster scenarios. |  |  |  |  |  |
| I am prepared to provide care during disasters, even when basic medications are unavailable. |  |  |  |  |  |
| I regularly review and update my knowledge of disaster preparedness measures. |  |  |  |  |  |
| I actively contribute to improving disaster preparedness within my healthcare facility. |  |  |  |  |  |
