# Supplementary material for: Evaluation of awareness, attitudes, and practices towards disaster preparedness among Saudi healthcare professionals: implications for enhancing emergency response and training
Source: PeerJ. 2025 Dec 15;13:e20464. doi: 10.7717/peerj.20464 (PMC12713557; doi:10.7717/peerj.20464)
Supplement: Supplemental Information 3 [file peerj-13-20464-s003.docx]

Supplementary Table - S1: Participants’ responses in the attitude section (n = 390). Responses are presented as frequencies and percentages across a 5-point Likert scale (Strongly agree to Strongly disagree).

| Attitude | Strongly agree  n (%) | Agree  n (%) | Neutral  n (%) | Disagree  n (%) | Strongly disagree  n (%) |
| --- | --- | --- | --- | --- | --- |
| I am keen to join disaster medicine education programs that focus on the country’s unique preparedness challenges | 83 (21.3) | 76 (19.5) | 81 (20.8) | 80 (20.8) | 70 (17.9) |
| I think healthcare professionals play a vital role in community disaster response | 61 (15.6) | 69 (17.7) | 80 (20.5) | 93 (23.8) | 87 (22.3) |
| I am open to taking on additional responsibilities related to disaster preparedness in my role. | 81 (20.8) | 86 (22.1) | 68 (17.4) | 81 (20.8) | 74 (19.0) |
| I believe disaster preparedness is important for healthcare professionals. | 82 (21.0) | 88 (22.6) | 53 (13.6) | 78 (20.0) | 89 (22.8) |
| I feel confident in my ability to respond effectively during a disaster. | 71 (18.2) | 81 (20.6) | 70 (19.0) | 94 (24.1) | 74 (19.0) |
| I believe that disaster preparedness training should be mandatory for healthcare professionals. | 76 (19.5) | 74 (19.0) | 60 (15.4) | 99 (25.4) | 81 (20.8) |
| I think my workplace adequately prioritizes disaster preparedness | 95 (24.2) | 76 (19.5) | 68 (17.4) | 74 (19.0) | 77 (19.7) |
| I believe that effective communication is crucial during a disaster. | 90 (23.1) | 80 (20.5) | 74 (19.0) | 87 (22.3) | 59 (15.1) |
| I feel supported by my organization in terms of disaster preparedness initiatives. | 86 (22.1) | 91 (23.3) | 74 (19.0) | 72 (18.5) | 67 (17.2) |
| I am willing to invest time and effort in ongoing disaster preparedness training. | 61 (15.6) | 63 (16.2) | 79 (20.3) | 88 (22.6) | 99 (25.4) |

Supplementary Table -S2: Participants’ responses in the practice section (n =390). Responses are presented as frequencies and percentages across a 5-point Likert scale (Strongly agree to Strongly disagree).

| Practice | Strongly agree  n (%) | Agree  n (%) | Neutral  n (%) | Disagree  n (%) | Strongly disagree  n (%) |
| --- | --- | --- | --- | --- | --- |
| I integrate disaster preparedness practices into my daily routine. | 74 (19.0) | 84 (21.5) | 80 (20.5) | 73 (18.7) | 79 (20.3) |
| I am confident in my ability to use first aid and CPR techniques during a disaster | 71 (18.2) | 69 (17.7) | 71 (18.2) | 98 (25.1) | 81 (20.8) |
| I actively contribute to disaster recovery planning efforts in my department | 90 (23.1) | 79 (20.3) | 65 (16.7) | 86 (22.1) | 70 (17.9) |
| Through sufficient attendance in disaster management training sessions, I feel ready to respond effectively during actual emergencies | 74 (19.0) | 91 (23.3) | 77 (19.7) | 69 (17.7) | 79 (20.3) |
| I feel comfortable executing evacuation protocols in the event of a disaster | 84 (21.5) | 88 (22.6) | 46 (11.8) | 86 (22.1) | 86 (22.1) |
| I am familiar with the emergency communication systems in place at my healthcare facility | 60 (15.4) | 99 (25.4) | 79 (20.6) | 88 (22.6) | 64 (16.4) |
| The training I received during my college courses has equipped me to function effectively in disaster scenarios. | 83 (21.3) | 83 (21.3) | 54 (13.8) | 90 (23.1) | 80 (20.5) |
| I am prepared to provide care during disasters, even when basic medications are unavailable. | 89 (25.1) | 85 (21.8) | 91 (23.3) | 57 (14.6) | 68 (17.4) |
| I regularly review and update my knowledge of disaster preparedness measures. | 98 (25.1) | 78 (20.0) | 56 (14.4) | 84 (21.5) | 74 (19.0) |
| I actively contribute to improving disaster preparedness within my healthcare facility. | 81 (20.8) | 77 (19.7) | 48 (12.3) | 96 (24.6) | 88 (22.60 |
